# Post–Acute Care Rehabilitation Services and Outcomes in Skilled Nursing Facilities Before and During the COVID-19 Pandemic

**DOI:** 10.1001/jamahealthforum.2023.0019

**Published:** 2023-03-03

**Authors:** Sandra M. Shi, Cyrus M. Kosar, Natalia Gouskova, Sarah Berry

**Affiliations:** 1 Hinda and Arthur Marcus Institute for Aging Research, Hebrew SeniorLife, Boston, Massachusetts; 2 Beth Israel Deaconess Medical Center, Department of Medicine, Harvard Medical School, Boston, Massachusetts; 3 Brown School of Public Health, Providence, Rhode Island

## Abstract

This cohort study evaluates changes in rehabilitation services provided by skilled nursing facilities during the COVID-19 pandemic.

## Introduction

The COVID-19 pandemic presented unprecedented challenges to skilled nursing facilities (SNFs), which provide post–acute care for nearly 20% of hospitalized older adults.^1^ To what degree SNFs delivered rehabilitative services while coping with stressors of the pandemic has not been previously explored, to our knowledge. The goal of this study was to characterize changes in rehabilitation services provided by SNFs during the pandemic.

## Methods

This retrospective cohort study used a large multistate data-sharing collaboration, representing 108 522 individuals in 776 nursing homes, and compared prepandemic (October 1, 2019-March 1, 2020) and pandemic (October 1, 2020-March 1, 2021) periods. These periods both occur after implementation of the Patient Driven Payment Model (PDPM), which began October 1, 2019, and match in calendar cycle to account for potential seasonal variations in case mix. Included SNFs were comparable to SNFs nationally (eTable in Supplement 1). This study population included all new short-stay admissions, defined as individuals not receiving care in the same facility within the past 180 days, with total length of stay less than 100 days. The Minimum Data Set, an administrative data set completed by trained nurses, was used to obtain demographic and clinical covariates (eg, race and ethnicity, Cognitive Function Scale [CFS] score,^2^ and Changes in Health, End-Stage Disease, Signs and Symptoms [CHESS] mortality risk score)^3^ and study outcomes, including therapy use (overall use, intensity, and duration of physical therapy [PT], occupational therapy [OT], and therapy for speech-language pathology [SLP]), community discharge, hospitalization, and mortality. All assessments and recorded minutes were included.

Linear probability models were used with SNF fixed effects, using a single model with a pre-post pandemic indicator for each outcome, accounting for potential changes in case mix. Estimated effects represent the adjusted effect estimate of the pandemic on rehabilitation intensity within a facility. Rehabilitation characteristics, including therapy days, minutes, and group setting outcomes, were estimated only among individuals who received the therapy. Analyses were done in Stata, version MP 16.0 (StataCorp LLC). A 2-sided significance threshold of *P* < .05 was used. The Brown institutional review board exempted this study, which followed the STROBE reporting guideline. Informed consent was waived because the data were deidentified.

## Results

The prepandemic and pandemic cohorts consisted of 61 017 and 47 505 patients, respectively (Table 1). Compared with the prepandemic cohort, the pandemic cohort had greater cognitive impairment, as indicated by CFS scores (61.6% vs 58.9% cognitively intact), and higher baseline mortality risk, as indicated by CHESS scores. Unadjusted differences in rehabilitation intensity prepandemic vs during the pandemic found fewer days of therapy (PT, 5.35 vs 4.86; OT, 5.17 vs 4.71; SLP, 4.01 vs 3.62) and fewer minutes per day of PT (47.87 vs 43.93) and OT (47.57 vs 44.07) but not SLP (34.77 vs 34.62) (Table 2). In adjusted analyses controlling for potential facility effect, differences remained significant and consistent with unadjusted analyses. The proportion of patients receiving group therapy during the pandemic decreased significantly (PT, 31.75% vs 3.49%; OT, 29.05% vs 3.16%; SLP, 11.13%vs 1.78%), though mean minutes of group therapy was low before the pandemic.

**Table 1.** Cohort Characteristics Prepandemic vs During the Pandemic^a^

Characteristic	Cohort, No. (%)	
	Prepandemic (n = 61 017)	Pandemic (n = 47 505)
Age at admission, median (IQR)	76.9 (67.9-85.1)	76.7 (67.7-84.7)
Sex		
Male	26 505 (43.4)	21 358 (45.0)
Female	34 512 (56.6)	26 147 (55.0)
Race and ethnicity		
American Indian or Alaska Native	163 (0.3)	164 (0.3)
Asian	1301 (2.1)	778 (1.6)
Black	9319 (15.3)	7058 (14.9)
Hispanic	1367 (2.2)	1082 (2.3)
White	45 780 (75.0)	35 637 (75.0)
Other^b^	3087 (5.0)	2786 (5.9)
Morris ADL score, median (IQR)	18.0 (14.0-19.0)	18.0 (15.0-20.0)
CFS score		
Intact (1)	36 657 (61.6)	27 185 (58.9)
Impairment		
Mild (2)	13 164 (22.1)	10 600 (23.0)
Moderate (3)	8063 (13.6)	6715 (14.6)
Severe (4)	1602 (2.7)	1622 (3.5)
CHESS Scale score^c^		
0	26 901 (49.1)	18 641 (44.7)
1	17 092 (31.2)	13 153 (31.5)
2	9666 (17.6)	8652 (20.8)
3-5	1108 (1.8)	1250 (2.6)
Comorbid conditions		
Dementia	10 998 (18.0)	8636 (18.2)
Atrial fibrillation or other dysrhythmias	16 258 (29.7)	13 052 (30.2)
Coronary artery disease	15 336 (25.1)	11 702 (24.6)
Heart failure	15 374 (25.2)	11 783 (24.8)
Hypertension	46 482 (76.2)	36 408 (76.6)
Hyperlipidemia	31 064 (50.9)	25 035 (52.7)
Diabetes	22 977 (37.7)	18 719 (39.4)
Urinary tract infection	7948 (13.0)	6664 (14.0)
Pneumonia	5020 (8.2)	4490 (9.5)
Septicemia	3982 (6.5)	3333 (7.0)
Chronic disease		
Lung^d^	16 982 (27.8)	12 341 (26.0)
Kidney^e^	18 063 (29.6)	14 945 (31.5)
Stroke/TIA	8100 (13.3)	6356 (13.4)
Hip fracture	5063 (8.3)	3932 (8.3)

Abbreviations: ADL, activities of daily living; CFS, Cognitive Function Scale; CHESS, Changes in Health, End-Stage Disease and Symptoms and Signs; TIA, transient ischemic attack.

^a^ The data for this table were obtained from 776 nursing homes in 41 states. States that did not contribute data were Alaska, Connecticut, Maine, Massachusetts, New Hampshire, New York, Oklahoma, Rhode Island, and Vermont.

^b^ Other includes unknown race, more than 1 race, or Native Hawaiian or Pacific Islander.

^c^ The CHESS scale indicates the degree of health instability. Higher scores indicate greater health instability.

^d^ Classified in the Minimum Data Set as “asthma, chronic obstructive pulmonary disease, chronic bronchitis, and restrictive lung diseases.”

^e^ Classified in the Minimum Data Set as “renal insufficiency, renal disease, or end-stage renal disease.”

**Table 2.** Comparison of Rehabilitation Services Provided and Other Outcomes Between the Prepandemic and Pandemic Periods

Outcome	Estimates					
	Unadjusted			Adjusted^a^		
	Mean		Difference (95% CI)	Mean		Difference (95% CI)
	Prepandemic	Pandemic		Prepandemic	Pandemic	
Total length of stay	36.24	37.89	1.64 (1.11 to 2.17)	38.8	38.87	0.07 (−0.41 to 0.55)
**Speech therapy** ^b^						
Received any therapy, %	36.52	37.46	0.95 (−0.32 to 2.21)	37.52	36.62	−0.91 (−2.11 to 0.30)
No. of days received	4.01	3.62	−0.39 (−0.49 to −0.30)	4.10	3.60	−0.50 (−0.59 to −0.41)
Minutes of therapy per day received	34.77	34.62	−0.14 (−0.64 to 0.35)	34.67	34.88	0.21 (−0.23 to 0.65)
Individual	33.76	34.51	0.75 (0.25 to 1.24)	33.63	34.74	1.10 (0.66 to 1.55)
Group	1.01	0.11	−0.89 (−1.00 to −0.78)	1.03	0.14	−0.89 (−1.02 to −0.77)
Received therapy in group setting at any time, %	10.95	1.55	−9.40 (−10.55 to −8.24)	11.13	1.78	−9.35 (−10.54 to −8.17)
Share of therapy in group setting, %	2.73	0.35	−2.39 (−2.68 to −2.09)	2.80	0.41	−2.39 (−2.72 to −2.05)
**Occupational therapy** ^b^						
Received any therapy, %	94.79	93.97	−0.81 (−1.33 to −0.29)	95.52	95.21	−0.30 (−0.77 to 0.16)
No. of days received	5.17	4.71	−0.46 (−0.56 to −0.37)	5.29	4.73	−0.56 (−0.64 to −0.47)
Minutes of therapy per day received	47.57	44.07	−3.50 (−4.02 to −2.98)	47.32	44.77	−2.56 (−3.04 to −2.08)
Individual	44.70	43.86	−0.84 (−1.38 to −0.31)	44.35	44.5	0.14 (−0.36 to 0.65)
Group	2.87	0.22	−2.66 (−2.88 to −2.44)	2.97	0.27	−2.70 (−2.93 to −2.48)
Received therapy in group setting at any time, %	28.12	2.74	−25.39 (−27.07 to −23.71)	29.05	3.16	−25.89 (−27.60 to −24.17)
Share of therapy in group setting, %	5.71	0.50	−5.21 (−5.61 to −4.80)	5.92	0.59	−5.33 (−5.77 to −4.90)
**Physical therapy** ^b^						
Received any therapy, %	95.38	94.93	−0.45 (−0.86 to −0.04)	96.07	96.20	0.13 (−0.23 to 0.49)
No. of days received	5.35	4.86	−0.49 (−0.58 to −0.40)	5.49	4.89	−0.60 (−0.68 to −0.51)
Minutes of therapy per day received	47.87	43.93	−3.94 (−4.45 to −3.43)	47.61	44.65	−2.97 (−3.45 to −2.49)
Individual	44.83	43.7	−1.13 (−1.68 to −0.58)	44.46	44.37	−0.09 (−0.61 to 0.43)
Group	3.04	0.23	−2.81 (−3.03 to −2.58)	3.15	0.28	−2.88 (−3.11 to −2.64)
Received therapy in group setting at any time, %	30.64	3.12	−27.51 (−29.32 to −25.71)	31.75	3.49	−28.26 (−30.09 to −26.42)
Share of therapy in group setting, %	6.01	0.54	−5.48 (−5.91 to −5.05)	6.26	0.60	−5.66 (−6.11 to −5.21)
**Secondary outcomes, %**						
Community discharge	68.92	65.36	−3.56 (−4.37 to −2.75)	70.40	69.41	−1.00 (−1.74 to −0.26)
Became long stay	12.19	13.76	1.57 (1.00 to 2.14)	13.37	13.94	0.57 (0.04 to 1.10)
General acute hospitalization						
Within 30 d of admission	14.65	15.38	0.73 (0.19 to 1.27)	11.73	11.77	0.04 (−0.48 to 0.55)
Within 100 d of admission	19.4	20.62	1.22 (0.62 to 1.83)	16.98	17.01	0.02 (−0.59 to 0.64)
Death						
Within 30 d of admission	2.53	3.15	0.62 (0.39 to 0.85)	2.07	2.12	0.05 (−0.16 to 0.26)
Within 100 d of admission	4.85	5.62	0.78 (0.47 to 1.09)	4.43	4.37	−0.05 (−0.35 to 0.25)
Community discharge or died within 100 d	73.71	70.94	−2.77 (−3.51 to −2.04)	74.78	73.73	−1.05 (−1.75 to −0.35)
Became long stay or died within 100 d	16.98	19.35	2.37 (1.69 to 3.06)	17.73	18.28	0.55 (−0.08 to 1.18)
Acute hospital or death						
Within 30 d of admission	16.97	18.29	1.32 (0.75 to 1.89)	13.67	13.73	0.06 (−0.50 to 0.61)
Within 100 d of admission	23.25	25.13	1.88 (1.24 to 2.53)	20.55	20.48	−0.06 (−0.73 to 0.60)

^a^ Adjusted point estimates are predictive margins derived from linear regression models with skilled nursing facility fixed effects. All regression models controlled for age, age-squared, female sex, race and ethnicity, activities of daily living impairment score, Cognitive Function Scale score, indicators for active diagnoses (dysrhythmia, coronary artery disease, heart failure, hypertension, hyperlipidemia, diabetes, urinary tract infection, pneumonia, septicemia, chronic lung disease, chronic kidney disease, stroke or transient ischemic attack, hip fracture, and dementia), Changes in Health, End-Stage Disease and Symptoms and Signs (CHESS) mortality risk score, and month of admission.

^b^ Therapy days, minutes, and group setting outcomes are estimated only among individuals who received the therapy.

## Discussion

This cohort study demonstrated that, during the pandemic, SNFs admitted patients with a greater burden of cognitive impairment and higher mortality risk. After adjusting for these case-mix changes, rehabilitation intensity declined modestly by roughly a half-day decrease in therapy across all 3 disciplines, approximately a 10% reduction from prepandemic levels. This finding may be partially explained by a large reduction in group therapy during the pandemic. These modest declines in rehabilitation intensity and community discharge during the pandemic were remarkable, as staffing levels decreased with higher turnover, leaving fewer staff present for a sicker patient population.^4,5^ Limitations of this study included using only a sample of SNFs, although overall characteristics remained fairly comparable to nationwide SNF characteristics. We were unable to adjust for potential changes in primary diagnoses, though we adjusted for conditions that may represent changes in case mix. Although we selected 2 time points that are post-PDPM, we were unable to distinguish between changes due to PDPM from changes due to the pandemic. In summary, despite exceptional challenges during the pandemic, SNFs were largely able to adapt and provide post–acute care rehabilitation services.

## Notes

Supplement 1. **eTable.** Comparison of study sampled skilled nursing facilities vs nationwide characteristics

Supplement 2. Data sharing statement
