# Error in Author Affiliations

**DOI:** 10.1001/jamahealthforum.2023.0748

**Published:** 2023-04-14

The Research Letter titled, “Post–Acute Care Rehabilitation Services and Outcomes in Skilled Nursing Facilities Before and During the COVID-19 Pandemic,”^1^ published online on March 3, 2023, included an error in the Author Affiliations section. Dr Kosar should only be affiliated with Brown University, and Drs Berry, Gouskova, and Shi should be affiliated with Hebrew SeniorLife and Beth Israel Deaconess Medical Center, Harvard Medical School. In addition, a middle initial for Sarah D. Berry was added. This error has been corrected.
