# Supplementary material for: Post–Acute Care Rehabilitation Services and Outcomes in Skilled Nursing Facilities Before and During the COVID-19 Pandemic
Source: JAMA Health Forum. 2023 Mar 3;4(3):e230019. doi: 10.1001/jamahealthforum.2023.0019 (PMC9984966; doi:10.1001/jamahealthforum.2023.0019)
Supplement: Supplement 1. — eTable. Comparison of study sampled skilled nursing facilities vs nationwide characteristics [file jamahealthforum-e230019-s001.pdf]

Supplemental Online Content

Shi SM, Kosar CM, Gouskova N, Berry S. Post–acute care rehabilitation services and outcomes in skilled nursing facilities before and during the COVID-19 pandemic. *JAMA Health Forum.* 2023;4(3):e230019.
doi:10.1001/jamahealthforum.2023.0019

eTable. Comparison of study sampled skilled nursing facilities vs nationwide characteristics

This supplemental material has been provided by the authors to give readers additional information about their work.

© 2023 Shi SM et al. *JAMA Health Forum.*

eTable. Comparison of study sampled skilled nursing facilities vs nationwide characteristics

**Characteristics, Median (IQR) or n (%)**	**Sample SNFs (n=776)**	**Other US SNFs (n=14790)**
Bed size	106.00 (73.00, 130.00)	99.00 (64.00, 126.00)
Occupancy rate	84.67 (72.93, 91.67)	83.80 (71.11, 91.67)
For-profit	442 (57.0%)	10480 (70.9%)
% Medicare financing	9.96 (5.05, 16.62)	9.52 (5.08, 16.20)
% Medicaid financing	63.59 (49.45, 75.38)	64.52 (48.08, 76.79)
Hospital-based	1 (0.1%)	644 (4.4%)
Direct care hours per day per resident*	3.46 (2.81, 3.74)	3.51 (3.03, 4.19)
Therapy hours per day per resident†		
Physical	0.17 (0.11, 0.25)	0.16 (0.10, 0.24)
Occupational	0.14 (0.10, 0.23)	0.14 (0.08, 0.20)
Speech	0.05 (0.02, 0.07)	0.04 (0.02, 0.06)
Overall star-rating		
1	103 (13.3%)	2361 (16.1%)
2	160 (20.7%)	2916 (19.9%)
3	165 (21.4%)	2722 (18.6%)
4	202 (26.2%)	3282 (22.4%)
5	142 (18.4%)	3349 (22.9%)
Census region		
Northeast	58 (7.5%)	2498 (16.9%)
Midwest	350 (45.1%)	4784 (32.3%)
South	196 (25.3%)	5271 (35.6%)
Pacific	172 (22.2%)	2237 (15.1%)
Rural	210 (27.1%)	4159 (28.1%)
COVID-19 burden		
Total resident infections per 100 beds during study period	18.58 (4.67, 43.11)	18.00 (3.73, 42.22)

© 2023 Shi SM et al. *JAMA Health Forum.*

Total staff infections per 100 beds during study period	18.52 (10.47, 32.17)	20.06 (10.48, 33.88)
Notes: IQR=Interquartile Range; SNF=Skilled Nursing Facility; US=United States; COVID-19= Coronavirus 2019 disease. SNF characteristics were obtained from Medicare's Care Compare website, the Certification and Survey Provider Enhanced Reports (CASPER) database, and the Payroll Based Journal PBJ). Rurality was classified using rural-urban continuum codes developed by the US department of agriculture, assigned to the county the SNF is located in. COVID-19 infection data were obtained from the Center for Disease Control and Prevention's National Healthcare Safety Network (NHSN) Long Term Care Facility Module database		
*Direct care hours are the sum of those provided by registered nurses, licensed practical nurses, and certified nursing assistants		
†Physical and speech therapy hours are the total of those provided by therapists, therapy assistants, and therapy aides. For speech therapy, only hours provided by therapists are reported in PBJ data		

© 2023 Shi SM et al. *JAMA Health Forum.*
