# Supplementary material for: Post–Acute Care Rehabilitation Services and Outcomes in Skilled Nursing Facilities Before and During the COVID-19 Pandemic
Source: JAMA Health Forum. 2023 Mar 3;4(3):e230019. doi: 10.1001/jamahealthforum.2023.0019 (PMC9984966; doi:10.1001/jamahealthforum.2023.0019)
Supplement: Supplement 2. — Data sharing statement [file jamahealthforum-e230019-s002.pdf]

Data Sharing Statement

Shi. Post-Acute Care Rehabilitation Services and Outcomes in Skilled Nursing Facilities Before and During the COVID-19 Pandemic. *JAMA Health Forum*. Published March 03, 2023. doi:10.1001/jamahealthforum.2023.0019

Data
Data available: No
